# Decoding the role of the nuclear receptor SHP in regulating hepatic stellate cells and liver fibrogenesis

**DOI:** 10.1038/srep41055

**Published:** 2017-01-24

**Authors:** Sabrina Cipriani, Adriana Carino, Dario Masullo, Angela Zampella, Eleonora Distrutti, Stefano Fiorucci

**Affiliations:** 1 Dipartimento di Medicina, Università degli Studi di Perugia, Nuova Facoltà di Medicina e Chirurgia, Sant’Andrea delle Fratte, Perugia, Italy; 2 Dipartimento di Scienze Chirurgiche e Biomediche, Università degli Studi di Perugia Nuova Facoltà di Medicina e Chirurgia, Sant’ Andrea delle Fratte, Perugia, Italy; 3 Dipartimento di Farmacia, Università di Napoli Federico II, Napoli, Italy; 4 Azienda Ospedaliera di Perugia, Perugia, Italy

## Abstract

The small heterodimer partner (SHP) is an orphan nuclear receptor that lacks the DNA binding domain while conserves a putative ligand-binding site, thought that endogenous ligands for this receptor are unknown. Previous studies have determined that SHP activation protects against development of liver fibrosis a process driven by trans-differentiation and activation of hepatic stellate cells (HSCs), a miofibroblast like cell type, involved in extracellular matrix (ECM) deposition. To dissect signals involved in this activity we generated SHP-overexpressing human and rat HSCs. Forced expression of SHP in HSC-T6 altered the expression of 574 genes. By pathway and functional enrichment analyses we detected a cluster of 46 differentially expressed genes involved in HSCs trans-differentiation. Using a isoxazole scaffold we designed and synthesized a series of SHP agonists. The most potent member of this group, ISO-COOH (EC_50_: 9 μM), attenuated HSCs trans-differentiation and ECM deposition *in vitro*, while in mice rendered cirrhotic by carbon tetrachloride (CCl_4_) or α-naphthyl-isothiocyanate (ANIT), protected against development of liver fibrosis as measured by morphometric analysis and expression of α-SMA and α1-collagen mRNAs. In aggregate, present results identify SHP as a counter-regulatory signal for HSCs transactivation and describe a novel class of SHP agonists endowed with anti-fibrotic activity.

Nuclear receptors (NRs) are a family of ligand-activated transcription factors^1^,^2^,^3^. Among the orphan members of the NR superfamily, the small heterodimer partner (SHP; NR0B2) has a unique structure that lacks the classical DNA-binding domain (DBD) while conserves a putative ligand-binding domain (LBD). SHP is a key transcriptional regulatory factor for a variety of genes that participate in diverse metabolic functions, including lipid and bile acid metabolism, as well as glucose homeostasis^1^,^2^,^3^. In these settings, SHP predominantly functions as a negative regulatory co-factor by interacting with other nuclear receptors, including the Farnesoid-x-receptor (FXR), Estrogen receptor (ER), hepatocyte nuclear factor (HNF) 4, androgen receptor (AR), Estrogen related receptor (ERR), liver receptor homolog-1 (LRH-1), liver X receptor (LXR), glucocorticoid receptor (GR), pregnane X receptor (PXR), retinoic acid receptors (RARs), RXR, thyroid hormone receptor (THR), and constitutive androstane receptor (CAR)^4^. In addition, SHP interacts with transcription factors, including c-Jun, SREBP-1c, and the forkhead transcription factor HNF3^5^.

SHP mRNA is predominantly expressed in the liver and at lower levels in heart and pancreas. It is also detected in the spleen and small intestine of human adults, in fetal liver, adrenal cortex and stomach^4^,^6^,^7^. SHP activation might have a role in protecting the liver from fibrosis. One early study showed that exposure of hepatic stellate cells (HSCs) to FXR ligands, induces SHP gene expression and reduced collagen1-α1 (Col1α1) and Transforming Growth Factor (TGF)-β1 expression by approximately 60–70% and abrogated Col1α1 mRNA up-regulation induced by thrombin and TGF-β1^8^. Further, fenofibrate, a PPARα agonist, increases SHP gene expression in the liver and protects from fibrosis induced by TGF-β1 and a methionine and choline-deficient diet through AMPK mediated induction of SHP gene expression^9^. Loss of SHP is also known to increase sensitivity to liver damage and fibrosis induced by bile duct ligation (BDL)^10^ establishing a major role for this gene in modulating transactivation of HSCs^11^.

Because its mechanistic role in liver fibrosis^8^,^11^, identification of SHP ligands and molecular pathways regulated by SHP may lead to discovery of new treatments for liver diseases. Consistent with this concept, SHP ligands were actively searched^12^,^13^. In order to identify new SHP target genes in HSCs we have generated stable clones of a rat and human HSC lines (HSC-T6 and LX2) overexpressing SHP^8^,^11^. By microarray analysis we have further characterized the HSC-T6 cell line and compared it to wild type HSC stimulated with chenodeoxycholic acid (CDCA), a FXR agonist. After pathway and functional enrichment analyses we have detected a cluster of differentially expressed genes involved in HSC transdifferentiation. Additionally, we report the design, synthesis and *in vitro* and *in vivo* characterization of a new class of SHP ligands based on the isoxazole scaffold that attenuates HSC trans-differentiation and development of fibrosis in a mice model of liver injury, highlighting a potential role for SHP ligands in the treatment of hepatic fibrosis.

## Materials and Methods

### Chemistry

#### General

Specific rotations were measured on a Jasco P-2000 polarimeter. High-resolution ESI-MS spectra were performed with a Micromass Q-TOF mass spectrometer. NMR spectra were obtained on Varian Inova 400 and Varian Inova 500 NMR spectrometers (^1^H at 400 MHz and 500 MHz, ^13^C at 100 MHz and 125 MHz) equipped with a Sun hardware and recorded in CDCl_3_ (δ_H_ = 7.26 and δ_C_ = 77.0 ppm) and CD_3_OD (δ_H_ = 3.30 and δ_C_ = 49.0 ppm). *J* are in hertz and chemical shifts (δ) are reported in ppm and referred to CHCl_3_ and CHD_2_OD as internal standards. HPLC was performed using a Waters Model 510 pump equipped with Waters Rheodine injector and a differential refractometer, model 401. Reaction progress was monitored via thin-layer chromatography (TLC) on Alugram^®^ silica gel G/UV254 plates. All chemicals were obtained from Sigma-Aldrich, Inc. Solvents and reagents were used as supplied from commercial sources with the following exceptions. Tetrahydrofuran, dichloromethane, diisopropylamine and triethylaminewere distilled from calcium hydride immediately prior to use. Methanol was dried from magnesium methoxide as follow. Magnesium turnings (5 g) and iodine (0.5 g) are refluxed in a small (50–100 mL) quantity of methanol until all of the magnesium has reacted. The mixture is diluted (up to 1 L) with reagent grade methanol, refluxed for 2–3 h then distilled under nitrogen. All reactions were carried out under argon atmosphere using flame-dried glassware. The purity of all of the intermediates, checked by ^1^H NMR, was greater than 95%. Detailed synthesis of compounds **1–7** is reported in Supplementary Figures 1 and 2) and Supplementary Materials and Methods.

### Animals

C57BL6 male mice were from Envigo (Udine, Italy) or from a C57BL6 colony hosted in the Perugia animal facility. The colonies were maintained in the animal facility of University of Perugia. Mice were housed under controlled temperatures (22 °C) and photoperiods (12:12-hour light/dark cycle), allowed unrestricted access to standard mouse chow and tap water and allowed to acclimate to these conditions for at least 5 days before inclusion in an experiment. A total number of 88 mice were used in this study. The study was conducted in agreement with the Italian and European guidelines. Experimental protocols were approved by a ethical committee of University of Perugia and by a National committee of Ministry of Health (permission n. 245/2013-B). The health and body conditions of the animals were monitored daily by the Veterinarian in the animal facility. The study protocol caused minor suffering, however, animals that lost more than 25% of the initial body weight were euthanized. At the day of sacrifice the mice were deeply anesthetized with a mixture of tiletamine hypochoride and zolazepam hypocloride/xylazine at a dose of 50/5 mg/Kg.

### Animal models

Liver fibrosis^8^ was induced in mice by carbon tetrachloride (CCl_4_) administration or α- naphthylisothiocyanate (ANIT) (see Supplementary Figures 4–6). For this purpose, C57BL6 male mice (22 animals) were administered i.p. 500 μL/Kg body weight of CCl_4_ dissolved in olive oil twice a week for 2 weeks. CCl_4_ mice were randomized, one week after of starting treatment, to receive compound **2** (ISO-COOH) (30 mg/Kg daily by gavage) or vehicle (1% methlylcellulose) for two additional weeks (Supplementary Figure 4). In a second set of experiments, C57BL6 male mice (24 mice) were administered i.p. 500 μL/Kg body weight of CCl_4_ dissolved in olive oil twice a week for 2 weeks. After one week, mice were randomized to receive CDCA (5 mg/kg daily by gavage) alone or in combination with compound **2** (ISO-COOH) (30 mg/Kg daily by gavage) or vehicle (1% methlylcellulose) for two additional weeks. Serum bilirubin, albumin and aspartate aminotransferase (AST) were measured by routine biochemical clinical chemistry. For histological examination, portions of the right and left liver lobes were fixed in 10% formalin, embedded in paraffin, sectioned and stained with Sirius red and Hematoxylin/Eosin (H&E) as described previously^8^,^11^. Additionally, samples obtained from each liver we stored for PCR analysis.

In a third set of experiments, C57BL6 male mice (42) were administered for 4 weeks with ANIT dissolved in olive oil (10 mg/kg per os) by gavage. After 2 weeks from the starting of ANIT treatment (See Supplementary Figure 6) mice were administered CDCA (5 mg/kg per os), or CDCA (5 mg/kg per os) plus ISO-COOH (30 mg/Kg per os), or ISO-COOH alone (30 mg/Kg per os) for 2 additional weeks (n = 9 per group). Control mice (n = 6) were treated with vehicles. At the end of the experiment, i.e. after 4 weeks treatment with ANIT, mice were sacrificed with anesthesia overdose and blood and liver were collected for biochemical analyses as described above.

### Rat and Human Hepatic Stellate Cell lines

HSC-T6, a rat immortalized HSC line, and LX2, an immortalized human HSC line^11^ were cultured at 37 °C in an atmosphere of 5% CO2 in Dulbecco’s modified Minimal Essential Medium (Gibco BRL Life Technologies, Rockville, MD, USA) containing 10% fetal bovine serum (FBS), 2 mM L-glutamine, and antibiotics (penicillin/streptomycin). Methods were carried out in accordance with the approved guidelines.

### Microarray analysis

The methods used for sample preparation, hybridization, data analysis, sensitivity and quantification were based on the Affymetrix GeneChip Expression Analysis Manual (Affymetrix, Santa Clara, CA, USA). In brief, HSC-T6, HSC-T6 stimulated 18 hours with 10 μM CDCA and HSC-T6 overexpressing SHP were used for the gene expression studies. Affymetrix Rat Genome 230 A GeneChip arrays containing more than 31,000 probe sets, analyzing over 30,000 transcripts and variants from over 28,000 well-substantiated rat genes were used. Further details can be obtained at http://www.affymetrix.com. Total RNA was extracted with the TRIzol Reagent (Invitrogen, Carlsbad, CA, USA). RNA was cleaned (RNeasy Mini Kit, Qiagen Inc., Valencia, CA, USA), converted to double-stranded cDNA (Gibco BRL Superscript Choice System, Life Technologies, Rockville, MD, USA) and then biotinylated to cRNA (Bioarray High Yield RNA Transcription Labeling Kit, Enzo Diagnostics, Farmingdale, NY, USA) according to the manufacturers’ protocols. Following fragmentation and quality control, the biotinylated cRNA was hybridized to the Affymetrix Rat Genome 230 A chip. Hybridized chips were washed, stained with streptavidin–phycoerythrin and scanned with a probe array scanner. GeneChip data were analysed by Affymetrix Microarray Suite MAS 5.0 software, which used one-sided Wilcoxon’s signed rank test to generate a ‘detection *P*-value’ (set at *P* < 0.05) to decide statistically whether a transcript was expressed on a chip. The detailed statistical analysis logic used to generate the ‘present/absent call’ and the ‘increase/decrease call’ is described at: http://www.affymetrix.comz/products/statistical_algorithms_reference_guide.html.

### Functional and pathway enrichment analysis

To perform functional enrichment tests of the candidate genes, we used g:Profiler software for Gene Ontology (GO) analysis. To investigate the pathways involving differentially expressed genes (DEGs), KEGG (Kyoto Encyclopedia of Genes and Genomes) pathway enrichment analysis was performed by using KOBAS (KEGG Orthology Based Annotation System) server based on cumulative hypergeometric distribution^14^. The criterion for this analysis was set as *p* value < 0.05.

### Validation of microarray data by custom PCR array

46 DEGs common between HSC-T6 stimulated with CDCA and HSC-T6 overexpressing SHP were validated using a custom RT^2^ profiler PCR array system (SAbiosciences). Array analysis was carried out with the online software RT^2^ Profiler PCR Array Data Analysis (http://pcrdataanalysis.sabiosciences.com/pcr/arrayanalysis.php).

### RNA extraction and Real-Time PCR

To investigate the effect of compound **2** (ISOCOOH) on hepatic stellate cell activity serum starved HSC-T6 and HSCT6 overexpressing SHP were stimulated 18 hours with 10 ng/mL TGF-β1 alone or in combination with 50 μM compound **2**. In another experimental setting serum starved LX2 and LX2 overexpressing SHP were stimulated 18 hours with 10 ng/mL TGF-β1 alone or in combination with 50 μM CDCA and 50 μM compound **2.** After treatments the relative mRNA expression of αSMA, α1collagen type 1 (COL1α1), and SHP was investigated by Real-Time PCR. Total RNA was isolated from cells or tissues using the TRIzol reagent according to the manufacturer’s specifications (Invitrogen). One microgram of purified RNA was treated with DNaseI and reverse transcribed with Superscript II (Invitrogen). For Real Time PCR, 10 ng cDNA were amplified in a 20 μL solution containing 200 nM of each primer and 10 μL of 2X SYBR FAST Universal ready mix (Invitrogen). All reactions were performed in triplicate, and the thermal cycling conditions were as follows: 10 min at 95 °C, followed by 40 cycles of 95 °C for 10 s, 55 °C for 10 s and 60 °C for 30 s in StepOnePlus instrument (Applied Biosystems). The relative mRNA expression was calculated and expressed as 2^−(ΔΔCt)^. Forward and reverse primer sequences were the following: rat GAPDH: atgactctacccacggcaag and tactcagcaccagcatcacc; rat SHP: cctggagcagccctcgtctcag and aacactgtatgcaaaccgagga; rat αSMA: gctccatcctggcttctcta and tagaagcatttgcggtggac; rat COL1α1: tgctgccttttctgttcctt and ggatttgaaggtgctgggta; human-GAPDH: gaaggtgaaggtcggagt and catgggtggaatcatattggaa; human-αSMA: acccacaatgtccccatcta and gaaggaatagccacgctcag; human-COL1α1: acgtcctggtgaagttggtc and cagggaagcctctctctcct; mouse-GAPDH: ctgagtatgtcgtggagtctac and gttggtggtgcaggatgcattg; mouse-TGFb1: ttgcttcagctccacagaga and tggttgtagagggcaaggac; mouse-COL1α1: acgtcctggtgaagttggtc and cagggaagcctctttctcct; mouse-αSMA: tgtgctggactctggagatg and gaaggaatagccacgctcag; mouse-TGR5: ggcctggaactctgttatcg and gtccctcttggctcttcctc; mouse-IL1β: tcacagcagcacatcaacaa and tgtcctcatcctcgaaggtc; mouse-FXR: tgtgagggctgcaaaggttt and acatccccatctctctgcac; mouse-SHP: tctcttcttccgccctatca and aagggcttgctggacagtta; mouse-BSEP: aaaacggatggtttcactgc and tgacagcgagtatcaccaag; mouse-OSTα: ctttggtgggaagaaagcag and gaagaaggcgtactggaaagg; mouse-NTCP: ggtgccctacaaaggcatta and gttgcccacattgatgacag; mouse-MCP1: cccaatgagtaggctggaga and tctggacccattccttcttg.

### SHP-GAL4 plasmid construction

To generate the fusion protein SHP-GAL4, the human ligand binding domain (LBD) of SHP was amplified by PCR from HepG2 genomic DNA and cloned SgfI/PmeI into the pFN26A (BIND) hRluc-neo Flexi Vector (Promega).

### Transactivation assay

HepG2 cells were maintained at 37 °C in E-MEM containing 10% FBS, 1% L-glutamine and 1% penicillin/streptomycin. The transfection experiments were performed using Fugene HD (Roche).

HepG2 cells were plated in a 6-well plate at 5 × 10^5^ cells/well. To evaluate the SHP transcriptional activity cells were transfected with 100 ng pFN26A-[SHPLBD/GAL4], 300 ng of the Promega reporter vector pGL4.35[luc2P/9XGAL4UAS/Hygro] and with 100 ng pGL4.70 (Promega), a vector encoding the human Renilla gene. 48 h post-transfection, cells were stimulated 18 h with 10 μM all trans retinoic acid ATRA, a SHP agonist, or with 10 μM compounds **1–7**. After treatments, cells were lysed in 100 μl diluted reporter lysis buffer (Promega) and 10 μl cellular lysate was assayed for luciferase activity using the Luciferase Assay System (Promega). Luminescence was measured with the Glomax 20/20 luminometer (Promega). Luciferase activities were normalized for transfection efficiencies by dividing the relative light units (RLU) by relative renilla units (RRU).

### Statistical analysis

All values are mean ± Standard Error (SE) of *number (n*) observations per group. Comparisons of more than two groups were made by one-way ANOVA with post-hoc Tukey’s test. The Student’s t-test for unpaired data was used when appropriate. The ANOVA test was used when more than 2 groups were analyzed. All tests were carried out using the GraphPad Prim (V5).

## Results

### Identification of differentially expressed genes (DEGs) and gene ontology (GO) enrichment analysis

**Figure 1:**
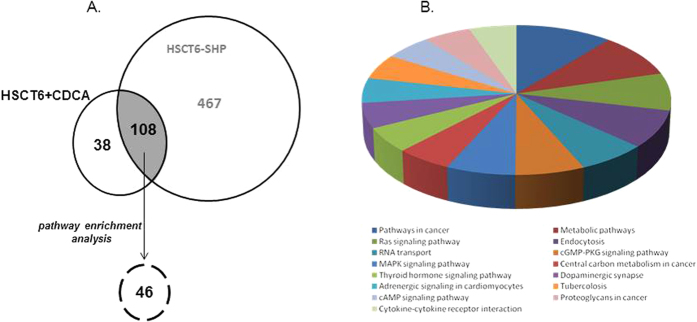
SHP overexpression or CDCA modulates common target genes in HSC. (**A**) Venn diagram of DEGs genes in HSC-T6 stimulated with CDCA and HSC-T6 overexpressing SHP. (**B**) Pathway analysis showing the top enriched canonical pathways. The enrichment of canonical pathways in the differentially expressed genes has been identified by KOBAS 2.0 software.

We have first conducted a gene expression analysis in HSC-T6 cells stimulated with CDCA and HSC-T6 cells overexpressing SHP by using the Affymetrix Microarray Suite MAS 5.0 software. This approach allowed the identification of 146 differentially expressed genes that were related to the effect of CDCA treatment and 575 genes that were related to SHP overexpression (Fig. 1A). Further analysis of these differentially expressed genes lead to the identification of 108 that were shared between the two cell lines (Fig. 1A). To explore whether DEGs share specific functional features, we performed a GO enrichment analysis by using the g:Profiler software. In term of GO database, the differentially expressed genes clustered into 3 main categories: Biological Process (BP), Cellular Component (CC) and Molecular Function (MF) (Table 1). The most significant terms in each of these three GO categories were: response to hormone in biological process (p value = 1.93 × 10^−8^), cell projection part in cellular component (p value = 3.95 × 10^−8^) and kinase binding in molecular function (2.99 × 10^−5^). Other enriched GO terms of interest include apoptotic process, striated muscle tissue development, intracellular signal transduction, regulation of phosphate metabolic process, mitotic cell cycle process, regulation of signaling and regulation of cellular component organization (Table 1).

### KOBAS pathway enrichment analysis

We used a public available tool for pathway analysis, the KOBAS 2.0, for finding enriched pathways in the 108 differentially expressed genes. The KOBAS 2.0 webserver uses KEGG Pathway, BioCyc, Reactome, Pathway Interaction Database and Panther databases to identify statistically enriched pathways in the differentially expressed genes (Entrez Gene IDs), against the background of all the genes in the human genome. We found that only 46 of the 108 DEGs were included in known enriched pathways including: cancer, Ras signaling, RNA transport, MAPK signaling, TR signaling, Adrenergic signaling, cAMP signaling, cytokine-cytokine receptor interaction, metabolic process, endocytosis, cGMP-PKG signaling, central carbon metabolism in cancer, dopaminergic synapse, proteoglycans in cancer and tubercolosis (Fig. 1B). 21 of these 46 DEGs showed the same fold of regulation between HSC-T6 stimulated with CDCA and HSC-T6 overexpressing SHP (i.e. NCoR1 and Rb1) (Table 2). By contrast, 25 of these 46 DEGs showed a different magnitude of regulation among HSC-T6 stimulated with CDCA and SHP-overexpressing HSC-T6 (i.e. CXCL12, TGFBR2, GR, DAG1). In particular, these genes were strongly downregulated in SHP-overexpressing HSC-T6 cells (i.e. TGFBR2: −2.26 times in HSC-T6 + CDCA and −12.2 times in HSC-T6 SHP) (Table 3). Of relevance, six of these 46 DEGs (CXCL12, TGFBR2, DAG1, GR, NCoR and Rb1) are mechanistically involved in the activation and trans-differentiation of HSC^15^,^16^,^17^,^18^,^19^,^20^,^21^.

### Validation of microarray by qRT-PCR analysis

**Figure 2:**
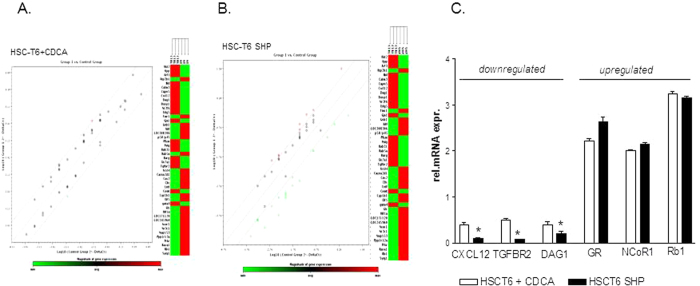
Microarray validation of gene array of SHP regulated genes using the RT^2^ *Profiler*™ PCR Array. (**A,B**) Scatter plot and clustergram of microarray data showing DEGs that are regulated by SHP (positively in red, negatively in green) in HSC-T6 stimulated with CDCA (A) or HSC-T6 overexpressing SHP (**B**). (**C**) Effect of CDCA treatment (white bars) and SHP over-expression (black bars) on the relative mRNA expression of CXCL12, TGFBR2, DAG1 GR, NCoR1 and Rb1.

The above mentioned results were validated by quantitative Real-Time PCR. To this end the 46 DEGs were amplified using a custom RT^2^ profiler PCR array system. Results in Fig. 2A and B showed highly significant concordance with microarray results for all 46 transcripts (Fig. 2A and B). In particular, while treatment of HSC-T6 cells with CDCA or SHP overexpression significantly reduced the relative mRNA expression of CXCL12, TGFBR2 and DAG1, the transcripts for GR, NCoR and Rb1 were upregulated (Fig. 2C, n = 3, p < 0.05). In aggregate these data demonstrate that SHP activation might have the potential to regulate HSC functions.

### Synthesis of isoxazole-based SHP agonist

Compounds **1**–**7** have been prepared as reported in Supplementary Figures 1 and 2. In detail, 2,6-dichlorobenzaldeide **8** was transformed in the corresponding hydroximic chloride **10** in turn prepared by chlorination of the intermediate **9**^22^. Condensation with oxime ethylisobutyryl acetate afforded **1** in 97% yield over three steps (Figure S1). Dibal-H reduction and LiOH hydrolysis at the ethyl ester group furnished the alcohol **3** and the carboxylic acid **2**, respectively. Compound **3** was also used as starting material for the synthesis of compounds **4**–**7**. Swern oxidation followed by Horner C2 homologation proceeded straightforward giving the conjugated ethyl ester **4** (84% yield over two steps, Figure S2), that in turn was transformed in the corresponding acid **5** by LiOH hydrolysis. In parallel, Dibal-H treatment on **4** furnished the allylic alcohol **6** that in a small aliquot was transformed in the C2 homologated ethyl ester 7 by Swern/Horner two steps sequence.

### SHP agonism reverts the pro-fibrotic phenotype in rat and human hepatic stellate cells

**Figure 3:**
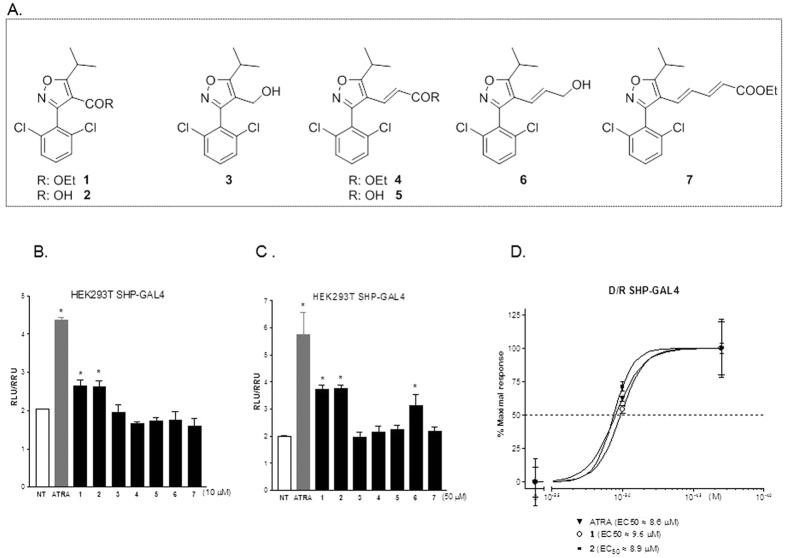
Identification and characterization of SHP agonists. (**A**) Molecular structures of compounds **1–7**. (**B,C**) Transactivation assay on SHP ligand binding domain. HepG2 cells were transiently transfected with the fusion protein SHP/GAL4 and with the reporter vector pGL4.35. 24 hours post-transfection Cells were stimulated with 10 μM (B) or 50 μM (**C**) compounds **1–7** and with 10 or 50 μM all trans retinoic acid (ATRA), used as a positive control. Results are expressed as the mean ± standard error (*p < 0.05 vs not treated cells (NT). (**D**) Concentration−response curves for compounds **1** and **2** (ISO-COOH). HepG2 cells transiently transfected with the fusion protein SHP/GAL4 and with the reporter vector pGL4.35 were stimulated with increasing concentrations of compounds **1** and **2** (1, 10 and 50 μM). ATRA (1, 10 and 50 μM) was used as a positive control to evaluate the SHP ligand binding domain activity.

To assess transcriptional activity, all the synthetic derivatives obtained in this study were tested in a luciferase reporter assays using HepG2 cells transfected with the fusion protein SHP/GAL4 and with the reporter vector pGL4.35[luc2P/9XGAL4UAS/Hygro] that contains nine tandem repeats of the GAL4 response element UAS. HepG2 cells were then stimulated with 10 or 50 μM compounds 1–7 or with all-trans retinoic acid (ATRA), a positive control in these experiments. As shown in Fig. 3A and B, compounds 1, 2 and 6 were SHP agonists in the transactivation assay. Further on, as shown in Fig. 3, compound 1 transactivated SHP with an EC_50_ of ~9.6 μM while compound 2 (ISO-COOH) induced SHP activity with an EC_50_ of ~8.9 μM. Both values were comparable to those obtained with the reference agonist ATRA (Fig. 3D; EC_50_ of ~8.6 μM).

**Figure 4:**
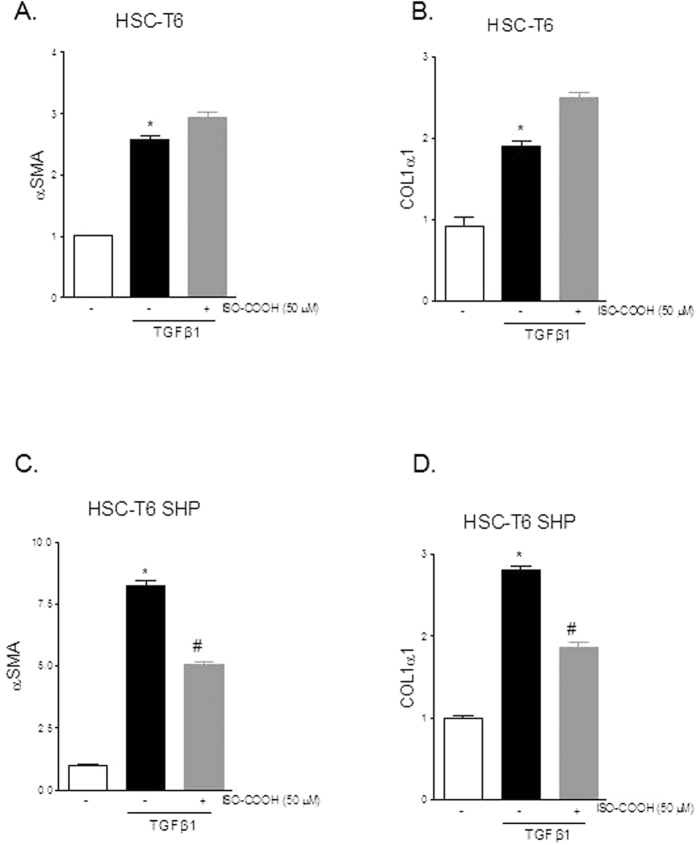
SHP agonism reverses the pro-fibrotic phenotype induces by TGFβ1 in rat HSC. Rat HSC-T6 and HSC-T6 overexpressing SHP were used. (**A,B**). Serum starved HSC-T6 were stimulated 18 hours with 10 ng/ml TGFβ1 alone or in combination with 50 μM ISO-COOH. At the end of stimulation the relative mRNA expression of αSMA and COL1α1 was assayed by Real-Time PCR. (**C,D**) Serum starved HSC-T6 overexpressing SHP were stimulated 18 hours with 10 ng/ml TGFβ1 alone or in combination with 50 μM ISO-COOH. At the end of stimulation the relative mRNA expression of αSMA and COL1α1 was assayed by Real-Time PCR. Values are normalized relative to GAPDH mRNA and are expressed relative to those of not treated cells (NT), which are arbitrarily set to 1. *p < 0.05 vs NT; ^#^p < 0.05 vs TGFβ1.

In the next set of experiments, we have examined the potential for compound **2** (ISO-COOH) to modulate HSC functions by assessing its effect on the expression of αSMA and COL1α1 in HSC-T6 and HSC-T6 SHP stimulated with 10 ng/ml TGFβ1. Results presented in Fig. 4 demonstrate that compound **2** significantly reduced the expression of αSMA and COL1α1 induced by TGFβ1 in HSC-T6 overexpressing SHP while it failed to do so in wild type HSC-T6 (Fig. 4A–D, n = 4, *p < 0.05 vs not treated cells, ^#^p < 0.05 versus TGFβ1 stimulated cells).

**Figure 5:**
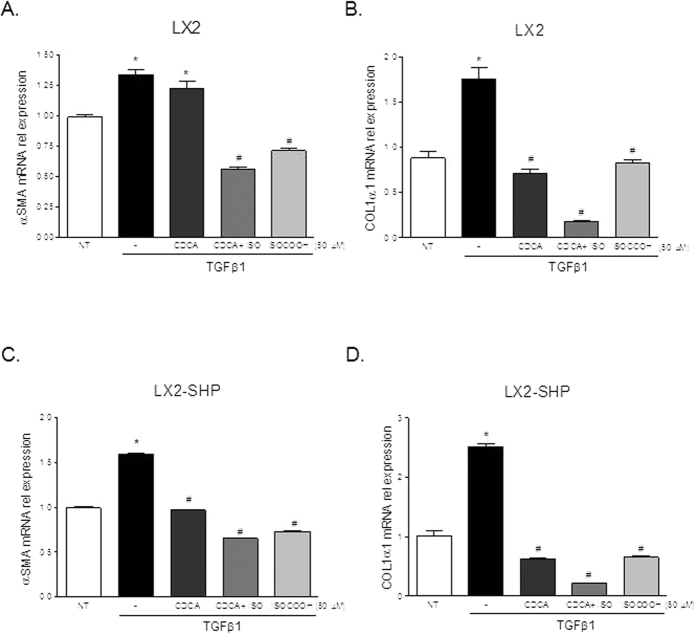
SHP agonism reverses the pro-fibrotic phenotype induced by TGFβ1 in human HSC. HSC-LX2 and HSC-LX2 overexpressing SHP were used. (**A,B**). Serum starved HSC-LX2 were activated with 10 ng/ml TGFβ1 and stimulated 18 hours with 50 μM CDCA and ISO-COOH 50 μM or with a combination of both agents. At the end of stimulation the relative mRNA expression of αSMA and COL1α1 was assayed by Real-Time PCR. (**C,D**) Serum starved HSC-LX2 overexpressing SHP were exposed to 10 ng/ml TGFβ1 for 18 h alone or in combination with 50 μM CDCA and 50 μM ISO-COOH or with a combination of both agents. At the end of the study cells were harvested and relative mRNA expression of αSMA and COL1α1 assayed by Real-Time PCR. Values were normalized relative to GAPDH mRNA and not treated cells (NT) were arbitrarily set to 1. *p < 0.05 vs NT; ^#^p < 0.05 vs TGFβ1.

We have then examined the effect of compound 2 in human HSC. Human HSC-LX2 and HSC-LX2 SHP were treated with 10 ng/ml TGFβ1 and after priming with CDCA 50 μM cells were treated with ISO-COOH alone. The administration of CDCA was necessary to activate FXR and thus increased the expression of his target gene SHP^8^,^11^. As shown in Fig. 5, ISO-COOH reduced the expression of αSMA and COL1α1 induced by TGFβ1 both in HSC-LX2 wild type and in HSC-LX2 overexpressing SHP (*p < 0.05 vs not treated cells, ^#^p < 0.05 versus TGFβ1 stimulated cells).

### SHP agonism protects against fibrosis development and accelerates fibrosis reversion in a mice model of liver fibrosis

**Figure 6:**
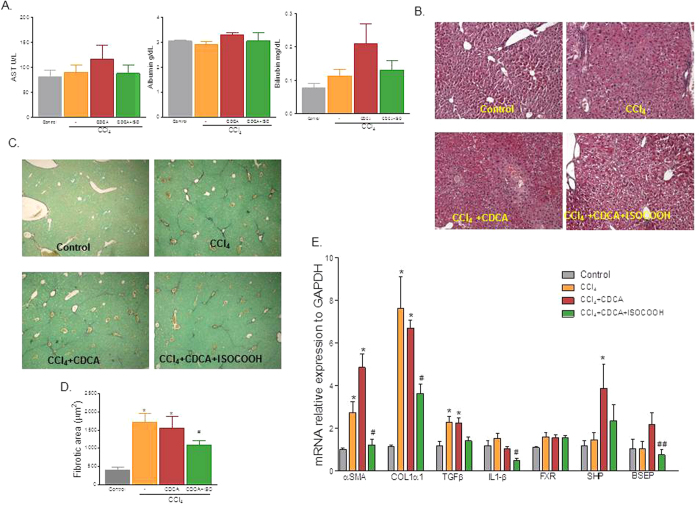
SHP activation protects against liver fibrosis in the CCL_4_ model. (**A**) Effect of compound 2 on AST, Albumin and Bilirubin in mice rendered cirrhotic by administration of CCl_4_. Mice were administered CCl_4_ for 3 weeks and after the first week of treatment randomized to receive CDCA, 5 mg/kg/day, alone or in combination with ISO-COOH, 30 mg/kg/day. (**B**) Hematoxylin and eosin (H&E) staining. (**C**) Syrius red staining. (**D**) Image J quantification of Syrius red staining. (**E**) Effect of CDCA (5 mg/kg) or ISOCOOH (30 mg/kg) on fibrotic genes in CCl_4_-treated mice. The relative hepatic mRNA expression of αSMA, COL1α1, TGFβ1, IL1β, FXR, SHP and BSEP was assayed by Real-Time PCR. Results are the mean ± SE of 4–6 mice per group. *p < 0.05 versus naïve mice. ^#^p < 0.05 versus CCl_4_ alone.

To investigate whether effects exerted by compound **2** were maintained *in vivo*, mice were administered with CCl_4_ for 2 weeks alone or in combination with ISO-COOH. The RT-PCR analysis shown in Supplementary Figure 3, demonstrated that ISO-COOH failed to reduce the relative mRNA expression of pro-fibrogenic markers including αSMA and COL1α1 in this setting. However, since CCl_4_ administration associates with a slight decrease of liver SHP mRNA expression^8^,^11^ these results were not surprising. Previous studies have shown CDCA, a weak FXR ligand, induces SHP expression in mice administered CCl_4_ without reducing liver fibrosis^11^. Thus, we designed a protocol (Supplementary Figure 4) in which CCl_4_ mice were administered with CDCA alone or in combination with ISO-COOH. Results of these experiments demonstrated that, mice administered CCl_4_ for 2 weeks developed a detectable liver injury (increased AST and bilirubin levels) and liver fibrosis (Sirius red and H&E staining) (Fig. 6A,B and C). While CDCA treatment worsened the liver injury, cotreating mice with CDCA and ISO-COOH reduced bilirubin levels. Morphometric analysis of liver section stained with H&E and Sirius red revealed that while a bridging fibrosis developed in CCl_4_-treated mice (Fig. 6, panels B, and C-D), these findings were reduced by treatment with CDCA in combination with ISO-COOH, but not by CDCA alone. Further on, as shown in Fig. 6E, RT-PCR analysis demonstrated that the CDCA treatment aggravated the fibrogenic condition, conversely the administration of ISO-COOH significantly reduced the expression of αSMA, COL1α1, TGFβ1 and IL1-β (n = 6, *p < 0,05 vs CTRL mice, ^#^p < 0,05 vs CCl_4_).

### SHP agonism protects against liver fibrosis development in a mice model of cholestasis

**Figure 7:**
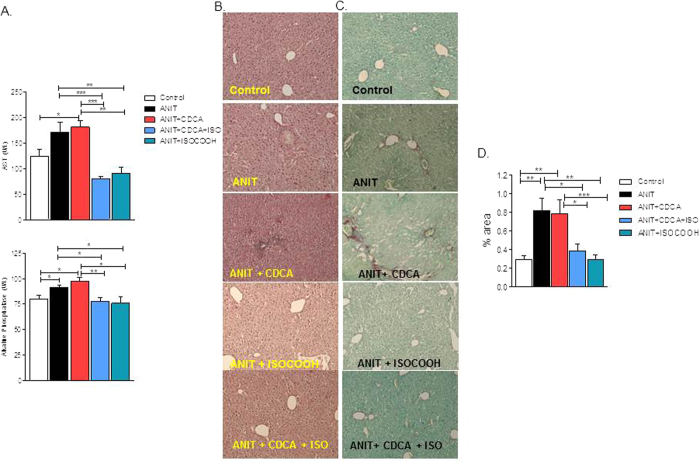
SHP agonism protects against liver fibrosis development in a mice model of cholestasis. (**A**) Effect of ISO-COOH on AST and Alkaline phosphatase in mice administered α-naphthyl-isothiocyanate (ANIT), 10 mg/kg per os for 4 weeks. Mice were randomized after 2 weeks to receive CDCA (5 mg/kg) alone or in combination with ISO-COOH (30 mg/Kg), or ISO-COOH alone (30 mg/Kg), for two additional weeks. Data shown are: (**B**) Hematoxylin and eosin (H&E) staining. (**C**) Syrius red staining. (**D**) Image J quantification of Syrius red staining. Results are the mean ± SE of 6–9 mice per group. *p < 0.05, **p < 0.005, ***p < 0.0005.

**Figure 8:**
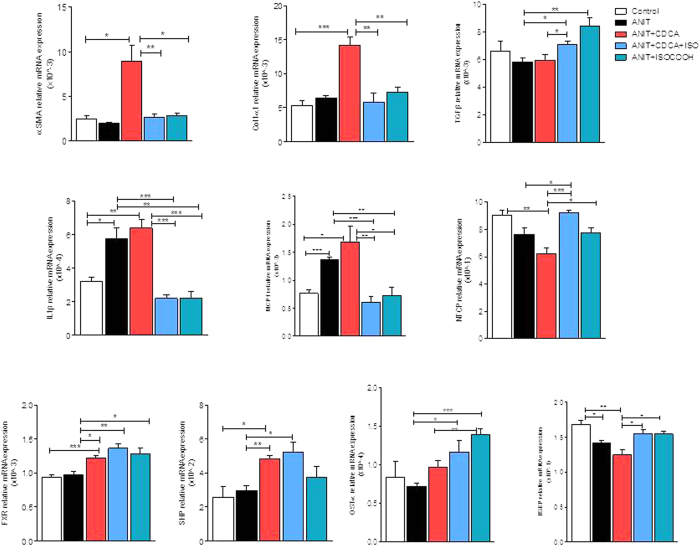
SHP activation protects against liver injury by modulating the expression of inflammatory genes. Effect of ISO-COOH on fibrosis, bile acid metabolism and inflammatory genes was assessed in mice administered α-naphthyl-isothiocyanate (ANIT), 10 mg/kg per os for 4 weeks. Mice were randomized after 2 weeks of ANIT to receive CDCA (5 mg/kg) alone or in combination with ISO-COOH (30 mg/Kg), or ISO-COOH alone (30 mg/Kg), for two additional weeks. The relative hepatic mRNA expression of αSMA, COL1α1, TGFβ1, IL1β, MCP1, NTCP, FXR, SHP, OSTα and BSEP was assayed by Real-Time PCR. Results are the mean ± SE of 6–9 mice per group. *p < 0.05, **p < 0.005, ***p < 0.0005.

Because the above mentioned data suggest that SHP activation by ISO-COOH accelerates the resolution of liver fibrosis in the CCl_4_ model only when administered in combination with CDCA, as a booster for SHP expression *in vivo*, we have designed a further investigation to dissect the relative contribution of these two agents in protecting against fibrosis development in mice exposed to ANIT. ANIT is xenobiotic whose administration triggers an immune responses leading to infiltration of mononuclear cells and periductular inflammation with injury of small intrahepatic bile ducts, bile duct regeneration and liver fibrosis. Treating mice with ANIT, resulted in liver injury and cholestasis as indicated by increased plasma levels of AST and alkaline phosphatase (Fig. 7A), and induced liver fibrosis as measured by morphometric analysis (Fig. 7C–D) and expression of αSMA and α1-collagen mRNAs (Fig. 8). Administration of CDCA alone failed to reverse this pattern, but administration of ISO-COOH either alone or in combination with CDCA protected against development of liver injury (Fig. 7A and B) and liver fibrosis (Fig. 7A,C and D, p < 0.05 versus ANIT alone and ANIT + CDCA). Consistent with these findings, ISO-COOH reversed the expression of αSMA and α1-collagen mRNAs induced by exposure of ANIT-treated mice to CDCA. Additionally, ISO-COOH, restored the expression of NTCP and BSEP and increased the expression of Ostα mRNAs, three FXR-regulated genes which are involved in bile acid uptakes and detoxification by hepatocytes. Finally, while administration of CDCA resulted in a slight increase of FXR and SHP expression, this agent had no effect on the expression of pro-inflammatory mediators such as IL-1β and MCP-1. In contrast, administration of ISO-COOH, alone or in combination with CDCA, reduced these pro-inflammatory mediators (Fig. 8). Taken together these data demonstrate that ISO-COOH protects against liver fibrosis and inflammatory changes in a model of liver injury and fibrosis caused by xenobiotic.

## Discussion

In the present study, we have used the transcriptional profiling of an immortalized hepatic stellate cell line (HSC-T6), over-expressing the orphan receptor SHP or stimulated with an FXR agonist, to identify both putative SHP target genes or intracellular pathways regulated by this orphan nuclear receptor. Thus, gene expression data were analyzed statistically to screen out DEGs, and pathway and functional enrichment analyses were performed to investigate the functional consistency of SHP target genes. Consequently, six candidate genes, that are involved in the activation and transdifferentiation of hepatic stellate cells, were identified including CXCL12, TGFBR2, DAG1, GR, NCoR, and Rb1. Supporting to these findings is the observation that SHP−/− mice showed a marked up-regulation of hepatic mRNA expression of TGFBR2, COL1α1 and αSMA (Figure S3).

Among these SHP regulated genes the chemokine CXCL12, together with its primary receptor CXCR4, play a major role during the progression of liver fibrosis by promoting hepatic stellate cell activation and contraction^23^. Furthermore, hepatic stellate cells express functional cell surface and intracellular CXCR4 and secrete high levels of CXCL12 allowing for both paracrine (from BECs and LSECs) and autocrine stimulation^18^.

TGFBR2 is the receptor for the transforming growth factor beta cytokines (TGFβ1, TGFβ2 and TGFβ3)^19^. Several works clearly demonstrated that TGFβ1, the most abundant isoform in both the normal and fibrotic liver, plays an important role in the regulation of the production, degradation and accumulation of extracellular matrix (ECM) proteins from HSCs, as well as in fibroproliferative changes that occur following tissue damage in liver fibrosis^19^,^24^.

DAG1 (Dystroglycan-1) is a membrane component of the dystrophin-glycoprotein transmembrane complex. Its expression is required for the spatial organization of laminin on the cell surface and for basement membrane assembly^16^. Several studies shows that dystroglycan is expressed on the membrane of hepatic stellate cells and is up-regulated by TGFβ1 and PDGF during liver fibrosis. The upregulation of DAG1 is implicated in the constitution of a perisinusoidal basement membrane during liver fibrosis^16^,^17^.

GR belongs to nuclear receptor superfamily of ligand activated transcription factors. It has been demonstrated that GR agonists (i.e. glucocorticoids, corticosteroids) interact with the TGF-beta signaling pathway on the transcriptional and translational level^20^. In particular, glucocorticoids decrease the bioavailability of TGFβ1, thus reducing hepatic stellate cell activation^20^.

Nuclear corepressor (NCoR) is a modular protein that contains three repressing domains in the N-termini and two domains that mediates interactions with nuclear receptors in the C-termini^25^. Previous published work demonstrated that a multiprotein complex consisting of NCoR and the heterodimer RAR/RXR suppresses COL1α1 expression in hepatic stellate cells upon the binding with retinoic acid (RA)^21^.

Retinoblastoma-1 (Rb1) is a classical tumor suppressor gene involved in the induction of apoptosis and blocking of cell cycle progression in many cellular types^26^. It has been reported that adenoviral transduction of Rb1 was effective to inhibit cell proliferation as well as to induce apoptosis in activated HSC^21^. Additionally, electron microscopic analysis confirmed that activation of the Rb mediated pathway in HSC results in chromatin and cytoplasmic condensation, typical features of ongoing apoptosis^26^.

Endogenous ligands for SHP have never been identified and SHP remains, bona fide, an orphan nuclear receptor. In the present study, we have modified the isoxazole scaffold and synthesized isoxazole derivatives, endowed with different side chains. Among the subsets of derivatives prepared in this study, 1 and 2 transactivate SHP with EC_50_ values of 9.6 and 8.9 μM, respectively. Both values are comparable to that of all trans retinoic acid (ATRA), a well documented SHP agonist.

The pharmacological properties of ISO-COOH have been further evaluated *in vitro*, using HSC-T6 and LX2 cells, two cell lines of rat and human origin. Both cell lines have been extensively used to characterize cell mechanism that govern hepatic fibrogenesis. The results of these experiments demonstrate that the isoxazole derivative, effectively reversed the pro-fibrogenetic phenotype caused by exposure of TGFβ1 only in SHP overexpressing HSC-T6 cells. In contrast, exposure of human LX2 to compound 2, resulted in a robust reduction of α-SMA and Col1α1 expression caused by exposure to TGFβ1. Importantly, the effect of the SHP agonist at least on Col1α1 was partially enhanced by coincubating the cells with CDCA (used as a SHP inducer). Further on, the anti-fibrotic activity of ISO-COOH, was slightly enhanced in SHP-overexpressing LX2 cells. Taken together, these results suggest that SHP agonism modulates pro-fibrogenetic activity of HSC and that these activities are conserved across species. However, the level of SHP expression in the target cells is critical, strongly supporting the notion that a combination therapy would be required to fully deploy the potential for ISO-COOH in the treatment of liver fibrosis.

To further investigate this concept, we have examined whether ISO-COOH protects against liver fibrosis induced in mice by CCl_4_, a well investigated model of liver fibrosis^8^. In these studies we found that treating mice, administered CCl_4_ for 2 weeks with ISO-COOH alone failed to protect against development of liver fibrosis as measured by assessing the liver content of αSMA and Col1α1 mRNAs (Supplementary Figure 3). In contrast, co-treating mice with CDCA to increase liver expression of SHP, resulted in a robust anti-fibrotic activity by ISO-COOH in this model (Fig. 6). Importantly, while CDCA alone had no effects on markers of liver fibrosis including αSMA, Col1α1 and TGFβ1 and worsened the severity of liver damage caused by CCl_4_, the combination of CDCA with ISO-COOH, resulted in a significant anti-fibrotic and anti-inflammatory activity. These findings were confirmed in a second model of liver fibrosis, induced by exposing mice to ANIT^27^,^28^. Treating mice with ANIT damages the cholangiocytes resulting in recruitment of inflammatory cells in the peri-duttular space, leading to bile duct proliferation and impaired bile flow^29^. This model of xenobiotic-induced cholestasis associates with progressive liver fibrosis that, in contrast to the CCl_4_ model, involves the activation of several types of myofibroblasts like cells^8^. In this model, ISO-COOH, effectively protected against development of liver injury and liver fibrosis and markedly attenuated changes in the liver expression of markers of inflammation^8^. The fact that SHP agonism was effective in reducing the liver fibrosis in the ANIT model, while required CDCA in the CCl_4_ model, is likely due to different pathogenic mechanisms involved in the two models and fibrosis severity.

In conclusion, the present study describes a new class of SHP agonists and identifies novel SHP regulated genes associated with the activation/transdifferentiation of HSCs, including CXCL12, TGFBR2, DAG1, GR, NCoR, and Rb1. In addition, we report the synthesis and pharmacological characterization of novel SHP agonists endowed with anti-fibrotic activity. The results of this study highlight the potential of SHP agonists in the treatment of liver fibrosis, however the fact that SHP agonism requires a FXR ligand to modulate the expression of pro-fibrogenetic genes in human LX2 cells, suggest that a combination therapy might be necessary in clinical settings.

## Additional Information

**How to cite this article**: Cipriani, S. *et al*. Decoding the role of the nuclear receptor SHP in regulating hepatic stellate cells and liver fibrogenesis. *Sci. Rep.* **7**, 41055; doi: 10.1038/srep41055 (2017).

**Publisher's note:** Springer Nature remains neutral with regard to jurisdictional claims in published maps and institutional affiliations.

## Supplementary Material

Supplementary Information

## Figures and Tables

**Table 1:** Gene Ontology (GO) terms enriched with module genes.

GO terms	P value	Observed	GO term ID
*Biological process*			
Striated muscle tissue development	0,0446	7	GO:0014706
Apoptotic process	0,000158	17	GO:0006915
Response to abiotic stimulus	0,000702	14	GO:0009628
Response to nutrient levels	0,0213	9	GO:0031667
Developmental process	7,63E-07	33	GO:0032502
Chemical homeostasis	0,0344	11	GO:0048878
Response to hormone	1,93E-08	18	GO:0009725
Regulation of localization	3,65E-08	24	GO:0032879
Positive regulation of VEGF production	0,0499	3	GO:0010575
Regulation of cardiac muscle tissue growth	0,0114	4	GO:0055021
Epithelial tube morphogenesis	0,0337	7	GO:0060562
Intracellular signal transduction	0,00856	17	GO:0035556
Regulation of phosphate metabolic process	0,000375	16	GO:0019220
Positive regulation of protein metabolic process	9,44E-06	17	GO:0051247
Reproductive process	0,00163	14	GO:0022414
Cell development	1,03E-05	20	GO:0048468
Multicellular organismal development	2,74E-06	30	GO:0007275
Rregulation of cell proliferation	0,000226	16	GO:0042127
Mitotic cell cycle process	0,00117	10	GO:1903047
Regulation of signaling	0,000807	20	GO:0023051
Reproduction	0,0279	11	GO:0000003
Growth plate cartilage chondrocyte growth	0,0118	2	GO:0003430
Regulation of cellular component organization	0,00383	17	GO:0051128
*Cellular component*			
Intracellular part	0,000859	42	GO:0044424
Cell projection part	0,000395	13	GO:0044463
Protein complex	0,0408	22	GO:0043234
Neuron projection	0,0135	12	GO:0043005
Membrane region	0,00746	12	GO:0098589
*Molecular function*			
Kinase binding	2,99E-05	12	GO:0019900

**Table 2:** Genes with the same fold regulation.

GENE	T6 + CDCA	T6SHP	Description	Accession	Gene ID
Acsl4	2,89	2,12	acyl-CoA synthetase long-chain family member 4	NM_053623	113976
Cacna2d1	3,12	4,58	calcium channel, voltage-dependent, alpha2/delta subunit 1	NM_012919	25399
Cav2	2,91	3,1	caveolin 2	NM_131914	363425
Cltc	2	2,99	clathrin, heavy polypeptide (Hc)	NM_019299	54241
Cntf	1,85	3,16	ciliary neurotrophic factor	NM_013166	25707
Comt	−2,72	−2,4	catechol-O-methyltransferase	NM_012531	24267
Cyp1b1	2,24	3,24	cytochrome P450, family 1, subfamily b, polypeptide 1	NM_012940	25426
Eif5	2,08	2,16	eukaryotic translation initiation factor 5	NM_020075	56783
gata4	−2,38	−3,39	GATA binding protein 4	NM_144730	54254
Gls	2,24	3,33	glutaminase	NM_001270786	192268
Hif1a	1,98	2,51	hypoxia inducible factor 1, alpha subunit	NM_024359	29560
LOC171120	2,13	2,96	Pr2 protein - Jmjd1c	NM_001191719	171120
LOC245960	2,53	2,4	potassium channel regulator 1 - Alg10	NM_139101	245960
Ncor1	1,85	2,2	nuclear receptor coactivator 1	NM_001271103	54299
Nr3c1	1,99	2,93	Glucocorticoid receptor	NM_012576	24413
Nup153	1,8	1,98	nucleoporin 153kD	NM_001100470	25281
Ppp1r12a	1,96	2,18	protein phosphatase 1, regulatory (inhibitor) subunit 12 A	NM_053890	116670
Prkr	2,18	3,06	Protein kinase, interferon-inducible double stranded RNA dependent -eif2ak2	NM_019335	54287
Rasa1	2,44	4,18	RAS p21 protein activator 1	NM_013135	25676
Rb1	2,72	3,23	retinoblastoma 1	NM_017045	24708
Synj1	1,73	2,18	synaptojanin 1	NM_053476	85238

**Table 3:** Genes with different fold regulation.

GENE	T6 + CDCA	T6SHP	Description	Accession	Gene ID
Akt2	−2,28	−8,35	murine thymoma viral (v-akt) oncogene homolog 2	NM_017093	25233
App	−2,15	−17	amyloid beta (A4) precursor protein	NM_019288	54226
Arf3	−2,51	−8,45	ADP-ribosylation factor 3	NM_080904	140940
Atp2b1	2,56	7,26	ATPase, Ca + + transporting, plasma membrane 1	NM_053311	29598
Bid	−2,26	−6,74	BH3 interacting domain death agonist	NM_022684	64625
Calm3	−2,26	−5,41	calmodulin 3	NM_012518	24244
Capn5	−2,02	−4,8	calpain 5	NM_134461	171495
Cxcl12	−2,52	−8,48	chemokine (C-X-C motif) ligand 12	NM_022177	24772
Dag1	−2,54	−5,27	dystroglycan 1	NM_053697	114489
Dusp1	−2,19	−23,5	dual specificity phosphatase 1	NM_053769	114856
Nr2f6	−2,61	−6,65	EAR-2	NM_139113	245980
Edg5	−2,41	−4,78	endothelial differentiation, sphingolipid G-protein-coupled receptor, 5 - sipr2	NM_017192	29415
Fmr1	2,88	5,03	fragile X mental retardation syndrome 1 homolog	NM_052804	24948
Gja1	−1,96	−6,75	gap junction membrane channel protein alpha 1	NM_012567	24392
Gnb1	2,58	5,47	guanine nucleotide binding protein, beta 1	NM_030987	24400
Kitl	2,74	5,87	kit ligand	NM_021843	60427
LOC308306	3,92	6,77	annexin V-binding protein ABP-7 - eif5b	NM_001110141	308306
p58/p45	1,94	4,22	nucleoporin p58 - nupl1	NM_139091	245922
Pfkm	−2,25	−4,91	phosphofructokinase, muscle	NM_031715	65152
Polg	−4,84	−12,9	polymerase (DNA directed), gamma	NM_053528	85472
Rab31	−2,41	−4,08	RAB31, member RAS oncogene family	NM_145094	246324
Rab5a	1,89	−2,21	RAB5A, member RAS oncogene family	NM_022692	64633
Rarg	−2,5	−8,12	retinoic acid receptor, gamma	NM_001135249	685072
Slc7a1	−2,43	−10,9	solutecarrierfamily7 (cationic amino acid transportery + system) member 1	NM_013111	25648
Tgfbr2	−2,26	−12,2	transforming growth factor, beta receptor II	NM_031132	81810
