# Supplementary material for: Decoding the role of the nuclear receptor SHP in regulating hepatic stellate cells and liver fibrogenesis
Source: Sci Rep. 2017 Jan 24;7:41055. doi: 10.1038/srep41055 (PMC5259793; doi:10.1038/srep41055)
Supplement: Supplementary Information [file srep41055-s1.doc]

**SREP-16-05947R1- Supplementary information**

Decoding the role of the nuclear receptor SHP in regulating hepatic stellate cells and liver fibrogenesis

Sabrina Cipriani1, Adriana Carino2, Dario Masullo3, Angela Zampella3,

Eleonora Distrutti4 and Stefano Fiorucci2

1Dipartimento di Medicina, Università degli Studi di Perugia, Nuova Facoltà di Medicina e Chirurgia, Sant’Andrea delle Fratte, Perugia, Italy

2 Dipartimento di Scienze Chirurgiche e Biomediche, Università degli Studi di Perugia Nuova Facoltà di Medicina e Chirurgia, Sant’ Andrea delle Fratte, Perugia, Italy

3 Dipartimento di Farmacia, Università di Napoli Federico II, Napoli, Italy

4Azienda Ospedaliera di Perugia, Perugia, Italy

**Supplementary Materials and Methods**

**Animals**

Livers from SHP null mice and their congenic littermates on C57B1/6 backgroundwere kindly provided by Dr. Li Wang (University of Connecticut, Storrs, Connecticut).

**3-(2,6-dichlorophenyl)-4-carboethoxy-5-isopropyl-isoxazole (1):** 1.2g compound **8** (5.6 mmol) was obtained starting from 1g of 2,6-dichlorobenzaldehyde (5.7 mmol) using a procedure with a 97.5% yield over three steps of reaction. Selected 1H NMR (400 MHz CDCl3): 7.4- 7.3 (3H, m),  4.1 (2H, dd, *J*=7.25), 3.88 (m),  1.44 (6H, d, *J*=6.9),  1.02 (3H, t, *J*=6.98).13C NMR (100 MHz CDCl3):  183.31,  161.08,  158.56,  135.4,  130.9 (2C),  127.84 (3C),  107.57,  60.44,  27.72,  20.16 (2C),  13.56. HR ESIMS *m/z* 328.05 [M+H]­+, C15H16Cl2NO3 requires 328.05.

**3-(2,6-dichloro-phenyl)-4-carboxyl-5-isopropyl-isoxazole acid (2):** Crude compound **1** (200 mg, 0.61mmol) was refluxed in *ca*. 50 mL of methanol-water 1:l with 30% NaOH. After stirring for 12 h, the basic aqueous solution was neutralized with HCl 6 N. Then methanol was evaporated and the residue was extracted with EtOAc (3 x 30 mL). The combined organic layers were washed with brine, dried and evaporated to dryness to give white solid residue, that was purified by silica gel chromatography, eluting with CH2Cl2 100% (153.8 mg, 84.3% yield). Selected 1H NMR (400 MHz CDCl3): 7.36-7.3 (3H, m), 3.85 (1H, m), 1.44 (6H, d, *J* = 6.95). 13C NMR (100 MHz CDCl3):  184.81,  166.34,  158.65,  135.33,  131.06 (2C),  127.81 (3C),  106.79,  27.82,  20.18 (2C). HR ESIMS *m/z* 298.0 [M-H]­-, C13H10Cl2NO3 requires 298.0.

**3-(2,6-dichlorophenyl)-4-hydroxymethyl-5-isopropyl-isoxazole (3):** a solution of 3-(2,6-dichlorophenyl)-4-carbomethoxy-5-isopropyl-isoxazole (800 mg, 2.45mmol) in tetrahydrofurane (10 ml) was cooled to 0°C under a nitrogen atmosphere while a solution of diisobutylammonium hydride (3.77 ml, 1.5M in toluene) was added dropwise. The reaction was allowed to warm slowly to ambient temperature over several hours and stirred at room temperature for 48h total. The flask was again cooled to 0° C and methanol (4ml) was carefully added over a 10 minutes period. Water (20 ml) was added dropwise and a gelatinous mixture formed. Sodium Hydroxide (30 ml, 2N) was added and the mixture was filtered over a plug of celite. After the solids were nearly dry, they were extracted with ethyl acetate (3 x 30ml) and the filtrates were combined. The organic layer was washed with water (2 x 30 ml) brine (30 ml), dried over anhydrous magnesium sulphate, filtered and condensed to give compound **3** (481.1 mg, 68.9% yield). An analytic sample was obtained by silica gel chromatography eluting with *n*-hexane: EtOAc97:3.Selected 1H NMR (400 MHz CDCl3):  7.45-7.35 (3H, m),  4.35 (2H, s),  3.35 (1H, m),  1.44 (6H, d, *J*=7.08). 13C NMR (100 MHz CDCl3):  176.27,  159.05,  135.69,  131.32 (2C),  128.16 (3C),  112.53,  53.63,  26.95,  20.9 (2C). HR ESIMS *m/z* 286.04 [M+H]+, C13H14Cl2NO2 requires 286.04.

**3-(2,6-dichlorophenyl)-4-ethylacrylate-5-isopropyl-isoxazole (4):** DMSO (994μl, 14mmol) was added dropwise for 5 min to a solution of oxalyl chloride (3.5 ml, 7mmol) in dry dichloromethane (5 ml) at −78°C under argon atmosphere. After 30 min, a solution of **3** (400 mg, 1.4mmol) in dry CH2Cl2 (5 ml) was added dropwise and the mixture was stirred at −78°C. After 30 min, Et3N dry (1.9ml, 14mmol) was addeddropwise to the solution and the mixture wasallowed to warm to room temperature. After 1 h, the reaction was quenched by addition of aqueous NaHSO4 (1 M, 50 ml). The layers were separated, and the aqueous phase was extracted with CH2Cl2 (3 × 30 mL). The combined organic layers were washed with saturated aqueous NaHSO4, saturated aqueous NaHCO3, and brine. The organic phase was then dried over Na2SO4 and concentrated to give the corresponding aldehyde (475 mg, quantitative yield) as a colorless oil, which was used without any further purification. To a solution of aldehyde (1.7mmol) in THF dry (10 ml) was added LiOH (93.7 mg, 3.9mmol) and TEPA (triethylphosphonoacetate, 742μl, 3.9mmol). The reaction mixture was stirred for 45 min at room temperature and then quenched with water (10 ml). The mixture was then extracted with EtOAc (3 × 30 mL), and the organic phase was concentrated in vacuo. Silica gel chromatography (CH2Cl2 100%) afforded pure **4** (415.2 mg, 84% over two steps). Selected 1H NMR (400 MHz CDCl3):  7.45-7.35 (3H, m),  7.38 (1H, d, *J*=16.1),  5.51 (1H, d, *J*=16.1),  4.17 (2H, dd, *J*=7.38),  3.43 (1H, m),  1.46 (6H, d, *J*=6.8),  1.27 (3H, t, *J*=6.94).HR ESIMS *m/z*354.07 [M+H]+, C17H18Cl2NO3 requires 354,07.

**3-(2,6-dichlorophenyl)-4-acrilic-5-isopropyl-isoxazole acid (5)**: A portion of compound **4** (100 mg, 0.3mmol) was hydrolyzed with lithium hydroxide (14.4 mg, 0.6mmol) in a solution of THF: H2O 1:1 v/v (5 mL). The resulting solution was then acidified with HCl 6N and extracted with ethyl acetate (3 x 30 mL). The collected organic phases were washed with brine, dried over Na2SO4 anhydrous and evaporated under reduced pressure to give **5** (85 mg, 92.8% yield). An analytic sample was obtained by silica gel chromatography eluting with CH2Cl2 100%.Selected 1H NMR (400 MHz CDCl3):  7.45-7.35 (3H, m),  7.41 (1H, d, *J*=16.1),  5.4 (1H, d, *J*=16.1),  3.43 (1H, m),  1.46 (6H, d, *J*=6.9).13C NMR (100 MHz CDCl3):  178.71,  171.63,  157.44,  133.57,  131.82 (2C),  128.42 (2C),  127.00 (3C),  116.92,  109.64,  27.0,  20.68 (2C). HR ESIMS *m/z* 324.02 [M-H]­-, C15H12Cl2NO3 requires 324.02.

**3-(2,6-dichlorophenyl)-4-prop-2-en-1-ol-5-isopropyl-isoxazole (6):** a solution of compound **4** (300 mg, 0.85mmol) in tetrahydrofurane (5 ml) was cooled to 0°C under a nitrogen atmosphere while a solution of diisobutylammonium hydride (2.8ml, 1.5M in toluene) was added dropwise. The reaction was allowed to warm slowly to ambient temperature over several hours and stirred at room temperature for 48h total. The flask was again cooled to 0°C and methanol (3 ml) was carefully added over a 10 minutes period. Water (10 ml) was added dropwise and a gelatinous mixture formed. Sodium Hydroxide (30 ml, 2N) was added and the mixture was filtered over a plug of celite. After the solids were nearly dry, they were extracted with ethyl acetate (3 x 50ml) and the filtrates were combined. The organic layer was washed with water (2 x 50 ml) brine (50 ml), dried over anhydrous magnesium sulphate, filtered and condensed to give compound **7** (200.9 mg, 76% yield). An analytic sample was obtained by silica gel chromatography eluting with CH2Cl2 100%.Selected 1H NMR (400 MHz CDCl3):  7.45-7.35 (3H, m),  7.35 (1H, d, *J*=16.1),  6.27 (1H, d, *J*=16.1),  4.13 (1H, d, *J*=5.89),  3.55 (1H, t, *J*=6),  3.22 (1H, m),  1.4 (6H, d, *J*=7.1).HR ESIMS *m/z* 312.06 [M+H]­+, C15H16Cl2NO2 requires 312,06.

**3-(2,6-dichlorophenyl)-4-(*E*)-ethyl penta-2,4-dienoate-5-isopropyl-isoxazole (7)**: DMSO (455μl, 1.6mmol) was added dropwise for 5 min to a solution of oxalyl chloride (1.6 ml, 6.4mmol) in dry dichloromethane (5 ml) at −78°C under argon atmosphere. After 30 min, a solution of **6** (100 mg, 0.32mmol) in dry CH2Cl2(5 ml) was added dropwise and the mixture was stirred at −78°C. After 30 min, Et3N dry (892μl, 6.4mmol) was addeddropwise to the solution and the mixture was allowed to warm to room temperature. After 1 h, the reaction was quenched by addition of aqueous NaHSO4 (1 M, 10 ml). The layers were separated, and the aqueous phase was extracted with CH2Cl2 (3 × 20 ml). The combined organic layers were washed with saturated aqueous NaHSO4, saturated aqueous NaHCO3, and brine. The organic phase was then dried over Na2SO4 and concentrated to give the corresponding aldehyde (150 mg, quantitative yield) as a colorless oil, which was used without any further purification. To a solution of aldehyde (0.46 mmol) in THFdry (5 ml) was added LiOH (24 mg, 1.02mmol) and TEPA (triethylphosphonoacetate, 175μl, 0.88 mmol). The reaction mixture was stirred for 30 min at room temperature and then quenched with water (5 ml). The mixture was then extracted with EtOAc (3 × 30 ml), and the organic phase was concentrated in vacuo. The solution was poured over a C18 silica gel column. Fraction eluted with *n*-hexane: EtOAc99:1 gave a mixture that was further purified by HPLC on a Nucleodur 100-5 C18 (5 mm; 4.6 mm i.d. x 250 mm) with MeOH/H2O (80:20) as eluent (flow rate 1 mL/min), to give 7.6 mg (6.25% yield, over two steps) of compound **7** (tR=16.2 min). Selected 1H NMR (400 MHz CDCl3):  7.48-7.39 (3H, m),  6.56 (2H, d, *J*=16.8),  5.94 (1H, t, *J*=14.8),  5.67 (1H, d, *J*=15.24),  4.19 (2H, dd, *J*=7.38),  3.37 (1H, m),  1.46 (6H, d, *J*=3.63),  1.29 (3H, t, *J*=7.1).HR ESIMS *m/z* 380.08 [M+H]­+, C19H20Cl2NO3 requires 380.08.

**Supplementary results**

**Supplementary Figures**

**Supplementary Figure 1. Synthesis of isoxazole derivative as SHP agonist.** Reagents and Conditions: a) NH2OH•HCl, NaOH in ethanol; b) N-chlorosuccinimide in DMF dry; c) ethyl isobutyryl acetate, sodium ethoxide, in THF dry, 97.5% yield.

**Supplementary Figure 2.** Synthesis of isoxazole derivative as SHP agonist. Reagents and Conditions: a) NaOH 5% in MeOH/H2O 1:1 v/v, 84.3 % yield; b) DIBAL-H in THF, Toluene, 68.9% yield; c) DMSO, oxalyl chloride, TEA dry, CH2Cl2, -78°C; d) LiOH, TEPA, THF dry, 84% over two steps; e) LiOH, THF: H2O 1:1 v/v, 92.8 % yield; f) DIBAL – H in THF, Toluene, 76 % yield; g) DMSO, oxalyl chloride, TEA dry, CH2Cl2, -78°C; h) LiOH, TEPA, THF dry, 84% over two steps.

**Supplementary Figure 3. Liver form mice that are SHP deficient have increased levels of pro-fibrogenetic genes.** (A) Effect of SHP knock-out on hepatic mRNA expression of TGFβ1, TGFβR2, CXCL12, COL1α1 and αSMA. (B) **C57BL6 mice were treated for 4 weeks with CCl4 alone or in combination with 50 μM compound 2** (30 mg/kg). The relative hepatic mRNA expression of αSMA and COL1α1 was assayed by Real-Time PCR. Results are the mean of 4 mice per group. *p<0.05 versus naïve mice.

**Supplementary Figure 4. Compound 2 fails to protect against development of liver fibrosis when administered alone.** (A) Experiment design: **C57BL6 mice were treated for 4 weeks with CCl4; one week of starting treatment, a group of 8 mice were treated with 50 μM compound 2** (30 mg/kg) for 2 weeks. (B) The relative hepatic mRNA expression of αSMA and COL1α1 was assayed by Real-Time PCR. Results are the mean of 8 mice per group. *p<0.05 versus naïve mice.

**Supplementary Figure 5. Compound 2, protects against development of liver fibrosis when administered in combination with a SHP inducer. Experimental design. C57BL6 mice were treated for 4 weeks with CCl4;** one week of starting treatment, mice were randomized to receive **CDCA (**5 mg/kg) alone or in combination with compound **2** (ISOCOOH) (30 mg/kg), for 2 weeks.

**Supplementary Figure 6. Compound 2, protects against development of liver fibrosis in the ANIT model. Experimental design. C57BL6 mice were treated for 4 weeks with ANIT. Two weeks after** starting the treatment, mice were randomized to receive **CDCA (**5 mg/kg) alone or in combination with ISOCOOH (30 mg/kg) ISOCOOH (30 mg/kg) for 2 weeks.
